# Compliance with Standard Precautions and Associated Factors among Healthcare Workers in Gondar University Comprehensive Specialized Hospital, Northwest Ethiopia

**DOI:** 10.1155/2017/2050635

**Published:** 2017-01-16

**Authors:** Tariku Gebre Haile, Eshetu Haileselassie Engeda, Abdella Amano Abdo

**Affiliations:** ^1^Department of Nursing, College of Medicine and Health Sciences, Wolkite University, Wolkite, Ethiopia; ^2^School of Nursing, College of Medicine and Health Sciences, University of Gondar, Gondar, Ethiopia; ^3^Department of Epidemiology and Biostatistics, College of Medicine and Health Sciences, Hawassa University, Hawassa, Ethiopia

## Abstract

*Background*. In many studies, compliance with standard precautions among healthcare workers was reported to be inadequate.* Objective.* The aim of this study was to assess compliance with standard precautions and associated factors among healthcare workers in northwest Ethiopia.* Methods.* An institution-based cross-sectional study was conducted from March 01 to April 30, 2014. Simple random sampling technique was used to select participants. Data were entered into Epi info 3.5.1 and were exported to SPSS version 20.0 for statistical analysis. Multivariate logistic regression analyses were computed and adjusted odds ratio with 95% confidence interval was calculated to identify associated factors.* Results.* The proportion of healthcare workers who always comply with standard precautions was found to be 12%. Being a female healthcare worker (AOR [95% CI] 2.18 [1.12–4.23]), higher infection risk perception (AOR [95% CI] 3.46 [1.67–7.18]), training on standard precautions (AOR [95% CI] 2.90 [1.20–7.02]), accessibility of personal protective equipment (AOR [95% CI] 2.87 [1.41–5.86]), and management support (AOR [95% CI] 2.23 [1.11–4.53]) were found to be statistically significant.* Conclusion and Recommendation.* Compliance with standard precautions among the healthcare workers is very low. Interventions which include training of healthcare workers on standard precautions and consistent management support are recommended.

## 1. Background

According to the literature, healthcare associated infections (HAIs) remain as the most frequent adverse event in any healthcare delivery system and affect millions of people each year, leading to significant morbidity and mortality [1]. Evidences throughout the literature showed that a large proportion of healthcare providers and clients had acquired infections within a healthcare facility [2–4]. In some studies, a mortality rate of up to 49% has been documented secondary to HAIs [5]. These infections, apart from posing very serious and life threatening conditions on healthcare workers and patients, are responsible for deterioration in the quality of healthcare and an increase in hospital costs [6, 7]. Data from American hospitals demonstrated that HAIs alone account for an estimated 1.7 million infections within a year. The same data also showed 98,987 HAIs associated deaths; of these, 36.3% were for pneumonia, 31% for blood stream infections, 13.2% for urinary tract infections, 8.3% for surgical site infections, and 11.2% for infections of other sites [8].

Although a well-established data were not available regarding the burden of HAIs in Africa, a systematic review done in the region revealed that its magnitude would be much higher than in the developed nations [9]. For instance, one Ethiopian study showed a high level of exposure to blood and body fluids among healthcare workers which put them at significant risk of HAIs [10]. Another Ethiopian study also revealed that significant numbers of healthcare workers (65.9%) were exposed to blood and body fluids, of which 29% were because of needle-stick injury [11]. Moreover, a point prevalence study in Ethiopia which was conducted on hospitalized patents identified 14.9% mean prevalence of hospital acquired infections [12]. A study done on postoperative patients also identified 10.9% confirmed bacterial nosocomial infections in Ethiopia [13].

According to the most recent guideline published by the Healthcare Infection Control Practices Advisory Committee (HICPAC) in 2007, it has been recommended to apply standard precautions (SPs) for all people during healthcare irrespective of their disease status. These SPs include but not limited to hand hygiene, use of personal protective equipment, and instrument processing [14]. In many studies, compliance with standard precautions among healthcare professionals was reported to be inadequate with regard to eye protection, avoidance of needle recapping, glove use when required, washing hands before and after patient contact, use of face masks, and avoidance of a used needle that is disassembled from a syringe and in implementation of precautions for all patients [15–17].

According to the literature, major reported factors that affect compliance with standard precautions include but not limited to lack of understanding and knowledge among healthcare workers on SPs [18, 19], shortage of time to implement the precautions (work overload), limited resources, lack of proper training, uncomfortable equipment, skin irritation, forgetfulness, distance from the necessary facilities, and insufficient support from management in creating a facilitating work environment [20]. Moreover, certain sociodemographic variables such as age, sex, job category, marital status, working site in the hospital and work experience were found to be associated with compliance with standard precautions [21, 22].

Interventions tried in other countries to increase the compliance of healthcare workers with SPs include but not limited to in-service training on SPs beyond ordinary level [23], preservice training by inclusion of SPs in educational curricula [24], and availability of personal protective equipment [6, 25].

Despite a significant improvement in facility of health institutions and in the number and kind of health task forces in Ethiopia particularly in the last two decades, local reports showed that there is still high burden of HAIs [11–13, 26]. On the other hand, except for some few studies on compliance of hand hygiene, very limited evidences are available with regard to the level of compliance of healthcare workers with standard precautions and its associated factors in the country in general and in the study area in particular. Therefore, this study was aimed at assessing the level of compliance towards standard precautions and its associated factors among healthcare workers in the Gondar University Comprehensive Specialized Hospital, Northwest Ethiopia.

## 2. Materials and Methods

An institution-based cross-sectional study was conducted from March 1 to April 30, 2014. The study was conducted in Gondar University Comprehensive Specialized Hospital, Northwest Ethiopia. Gondar town is located in the northern part of Ethiopia in Amhara Regional State, 748 km far from the capital city, Addis Ababa. The hospital, having 540 beds, is a tertiary level teaching and referral hospital which acts as a referral centre for district hospitals in the area and serves more than five million inhabitants.

All healthcare workers who were assigned to clinical services during the study period and had regular program for direct patient care including graduating class intern doctors were included in this study.

The outcome variable of the study was healthcare workers' compliance with standard precautions (always compliant). The independent variables of the study were sociodemographic factors (age, sex, marital status, job category, work experience, and assigned place), individual factors (knowledge about SPs, attitude towards SPs, risk taking personality, perception about efficacy of prevention, perception of risk, and peer influence), and institutional factors (safety equipment availability, safety equipment accessibility, management support for safe work practice, safety performance feedback, and work place safety climate).

Sample size was determined using single population proportion formula considering the following assumptions: 95% confidence interval, 5% margin of error, 50% proportion (since there was no previous study), and 10% nonresponse rate, and the final sample size was 423. Simple random sampling technique was used to select the study participants. The samples were proportionally allocated to each category of healthcare workers and respondents were selected using computer generated random number.

Data were collected using structured and self-administered questionnaire by four trained diploma nurses and the supervisors were two B.S. nurses (all working in another health facility). The questionnaire was developed based on the Ethiopian national infection prevention and patient safety guideline [27]. A pretest was conducted in a nearby hospital among healthcare workers (5% of the total sample size). Compliance with standard precautions was measured using 22 items on a 3-point Likert scale (1 = seldom, 2 = sometimes, and 3 = always).

Data quality was assured by training of data collectors and supervisors, pretesting the questionnaire on similar setting that was not included in the study, close supervision and assistance of data collectors, and checking filled questionnaires on daily basis for completeness, clarity, and accuracy of data.

Data were entered into Epi info 3.5.1 and were exported to SPSS version 20.0 for statistical analysis. At the beginning of the analysis, those who reported that they were always compliant were taken as “compliant” and those who reported that they were sometimes and seldom compliant were taken as “noncompliant.” Next, summation of the 22 compliance items was made. Then, the variable was recoded and dichotomized (compliant/noncompliant). Descriptive statistics were carried out to illustrate means, standard deviations, and frequencies of the study variables. Bivariate and multivariate logistic regression analyses were computed to identify variables having significant association with the dependent variable. Odds ratio with 95% confidence interval was used to determine the strength of association between dependent and independent variables. For this study all variables having *p* value of less than or equal to 0.2 were entered into multivariable logistic regression analysis. Variables having a *p* value of less than or equal to 0.05 in the multivariable logistic regression model were considered as significantly associated variables.

In this study, regularly employed hospital workers who were providing direct patient care and/or had direct contact with patients during their routine task and graduating class intern doctors were considered as healthcare workers. After summation of the 22 compliance items, participants who had scored a cut-off point of 66 (22 items times 3 points) were considered as “compliant” with standard precautions.

In this study “compliance with standard precautions” means the healthcare workers always adhere to all of the 22 components of standard precautions.

Ethical clearance was obtained from the ethical review committee of college of medicine and health sciences, University of Gondar. Permission was obtained from the clinical director of the study hospital. Informed consent was obtained from each study participant after explanation of how they would take part in the study and if any involvement was required after they completed the consent. Anyone not willing to participate in the study would have full right not to participate. All data collectors and the research team used code numbers rather than names and kept the questionnaires locked in order to ensure confidentiality. Healthcare workers who were exposed to potentially infected body fluids during the data collection period were provided with first aid and referred to infection prevention committee of the hospital for better management and follow-up.

## 3. Results

### 3.1. Sociodemographic Characteristics

Four hundred seven respondents returned completely filled questionnaires which gave a response rate of 96.2%, out of which 263 (64.6%) were males. The mean (SD) age of respondents was 27.9 (4.6) years. Two hundred fifty-five (62.7%) participants were single. The most frequent professional category was nurse 162 (39.8%), followed by intern doctor 80 (19.7%). The mean (SD) work experience was 3.7 (3.58) years ranging from 1 to 36 years (Table 1).

### 3.2. Level of Compliance with Standard Precautions

Among the healthcare workers who participated in this study 80.6%, 18.4%, and 39.6% reported that they always wash hands after removal of gloves, before touching patient, and before clean or aseptic techniques, respectively. Only 32.4% of the respondents reported that they always protect themselves against body fluid exposure regardless of the diagnosis of patients while 88.7% of HCWs reported that they always wear gloves whenever there is a possibility of exposure to any body fluids. The compliance of the HCWs with wearing a waterproof apron and eye goggles whenever there is a possibility of body fluid splashing and the compliance of HCWs in segregation of infections and noninfectious wastes into appropriate dust bins were found to be below 50% (Table 2).

In this study (by summing up individual items of the components of SPs and taking those who were always compliant as an outcome variable), the overall proportion of HCWs who were always compliant with SPs was found to be 12%. The most compliant healthcare workers were nurses (3.4%) followed by intern doctors and general practitioners (2.5%) (Table 3).

### 3.3. Factors Associated with Compliance with SPs

In the bivariate analysis sex, knowledge about SPs, perception of infection risk, received training on SPs, the availability and accessibility of PPE, management support, workplace safety climate, and feedback on safety practices were significantly associated with compliance. However, when these variables were analyzed together in the multivariate analysis using backward logistic regression method, only sex, perception of infection risk, training on SPs, accessibility of PPE, and management support were found to be statistically significant predictors of compliance.

In this study, female healthcare workers were 2.18 times more likely to be always compliant with standard precautions as compared to male HCWs. Healthcare workers who had taken infection prevention training were 2.9 times more likely to be always compliant with standard precautions as compared to nontrained HCWs. In addition to this, HCWs who had more frequent management support towards safety environment at the institution were 2.23 times more likely to be always compliant than those who had less frequent management support. Respondents of this study who had higher infection risk perception were 3.46 times more likely to be always compliant with SPs as compared to those who had lower infection risk perception. Moreover, HCWs who had readily accessed PPE were 2.87 times more likely to be always compliant than those who had not readily accessible PPE (Table 4).

## 4. Discussion

In developing counties like Ethiopia where resources are very limited, compliance with standard precautions is a cost effective strategy to prevent HAIs. This study showed the magnitude of healthcare workers compliance with SPs and sociodemographic, individual and institutional factors which affect compliance.

In this study, the overall compliance of HCWs with SPs was found to be very low (12%). However, when each of the specific components of SPs was analyzed, better results have been observed in some of the items. For instance, a relatively higher proportion of HCWs were found to be always compliant with washing hands after body fluid exposure (92.2%), washing hands immediately after removal of gloves (80.6%), wearing clean gloves whenever there is a possibility of exposure to body fluids (88.7%), changing gloves between contacts with different patients (88.9%), and placing used sharps in puncture-resistant container at point of use (87.2%). A relatively similar finding has been observed in one Nigerian study in which 82.6% of participants washed their hand after patient contact and 81.2% of the participants washed their hands after contact with contaminated equipment or surface. In line with our study, the Nigerian study has also revealed that 80% of HCWs who participated in the study disposed sharps immediately in puncture-resistant safety box [16]. On the contrary, a very low proportion of HCWs had reported that they were always compliant with washing hands before touching a patient (18.2%), providing nursing care considering all patients as potentially infectious (27%), avoiding wearing of their uniform outside the hospital compounds (37.6%), wearing goggles and/or masks whenever there is a possibility of body fluid splash in their face (21%), segregating noninfectious wastes in black color coded dust bin (30.2%), and segregating infectious medical wastes in yellow color coded dust bin (34.4%) in our study. These findings were consistent with other studies in that a lesser proportion of HCWs always comply with washing hands before patient contact [16], using face-mask, and changing uniform on exit [16, 17].

In spite of the fact that significant numbers of HCWs were always compliant with some of the components of SPs in the current study, it seems that they were protecting themselves in most cases. Therefore, one of the most important implications of these findings could be that the HCWs were not protecting patients, families, visitors, and the community at large from hospital acquired infections as per the recommended guidelines. On the other hand, higher proportions of HCWs were not always compliant with some other components of SPs (e.g., in considering all patients potentially infections and in using eye goggles and face masks whenever appropriate) which were mainly important to protect themselves (and also their families) from acquiring deadly infections. One possible explanation for these findings could be lack of up-to-date training on the principles of SPs. Moreover, unavailability of certain personal protective equipment (e.g., goggles and face masks) and inconsistent management support may also be additional potential reasons for the lower performance. On the contrary, the current study showed that 88.9% of HCWs were changing gloves between patients. This finding could strengthen the assumption that the noncompliance in other components might be due to unavailability of protective supplies.

In this study, female HCWs were more likely to always comply with SPs as compared to male HCWs. The female HCWs' better compliance might be due to a natural tendency of female workers to obey organizational rules and regulations most often and also to their extra caution against infections. Moreover, in Ethiopia since females take the lion's share in taking care of children and being responsible for cooking meals for their family, they might be more concerned about their health in order not to bring infectious disease to their family.

This study showed that HCWs who had higher perception of infection risk were more likely to always comply with standard precautions than those who had lower perception of risk. This finding could be explained by the fact that whenever HCWs have increased perception of exposure to infection, their level of adherence to preventive guidelines would increase.

In line with Chinese and Brazilian studies [6, 21], this study also revealed that HCWs who took training on standard precaution guidelines were more likely to always comply with SPs as compared to nontrained HCWs. The possible explanation for this finding could be the fact that training on current guidelines could upgrade the knowledge and skill of HCWs in that they would easily understand basic principles, recommendations, and standards of practice and implement them consistently whenever it is essential to do so. Moreover, up-to-date knowledge and skill regarding SPs could also increase the confidence of HCWs in complying with recommended guidelines.

In our study HCWs who had more frequent management support towards safety environment in their institution were more likely to always comply with SPs as compared to those who had less frequent support. A supporting result has been observed in a Brazilian study in that management support positively impacted compliance of HCWs with SPs [28]. This might be due to the fact that management bodies could play a key role and are responsible to make accessible all necessary safety equipment for those HCWs who need it and to build safe workplace safety climate for themselves, HCWs, and patients at large. It is also obvious that, without a management support and decision, it could be very difficult to renovate infrastructures suitable to infection control and it could hardly be possible to allocate sufficient budget for infection prevention activities. Moreover, management support could also increase the compliance of HCWs with SPs by recognizing role models and establishing a rewarding system for those who consistently implement recommended guidelines and policies. In addition to that, management support could also help strengthening infection prevention activities by designing controlling mechanisms and taking corrective measures on noncompliant HCWs.

In this study, HCWs who did not have readily accessible personal protective equipment were less likely to always comply with SPs as compared with those who had readily accessible personal protective equipment. This finding could be explained by the fact that almost all SPs require some kind of personal protective equipment which needs to be accessible at the point of use. On the other hand, unless a healthcare worker has a favorable attitude towards complying with SPs, he/she might take the absence of certain modalities and equipment as an advantage not to practice recommended guidelines. Moreover, frequent inaccessibility of personal protective equipment could decrease the motivation of previously energetic staff and could be a reason for noncompliance.

One of the major limitations of this study is its cross-sectional nature in which it does not establish definitive cause and effect relationships between the outcome and explanatory variables. On the other hand, since self-reported data had been used, the reliability of the results might be negatively influenced to some extent because of response bias.

## 5. Conclusion

Compliance with SPs among the HCWs of Gondar University Comprehensive Specialized Hospital is very low. Being female healthcare worker, higher perception of infection risk, accessibility of personal protective equipment, training on infection prevention, and management support were significantly associated factors with compliance of SPs. Training of HCWs on SPs, fulfilling infection prevention materials and equipment, and consistent and strong management support are recommended.

## Figures and Tables

**Table 1:** Sociodemographic characteristics of HCWs working in Gondar University Comprehensive Specialized Hospital, Northwest Ethiopia, 2014 (*n* = 407).

Variable	Category	Frequency	%
Age category	≤25 years	135	33.2
	26–30 years	207	50.9
	≥31 years	65	15.9
Sex	Female	144	35.4
	Male	263	64.6
Profession	Intern doctors	80	19.7
	General practitioners	48	11.8
	Anesthetists	14	3.4
	Nurse	162	39.8
	Midwifes	22	5.4
	Laboratory technologists	65	16.0
	Radiologists	5	1.2
	Physiotherapists	11	2.7
Work experience	≤2 years	165	40.5
	3-4 years	150	36.9
	5 years and above	92	22.6
Marital status	Single	255	62.7
	Married	141	34.6
	Widowed	4	1.0
	Divorced	7	1.7
Assigned place (ward)	OPD^*∗*^	44	10.8
	MDR TB ward	10	2.5
	Ophthalmology ward	13	3.2
	Fistula ward	6	1.5
	General ward	10	2.5
	Laboratory room	64	15.7
	Physiotherapy room	11	2.7%
	Radiology room	5	1.2%
	Emergency and inpatient	19	4.7%
	Medical ward	40	9.8%
	Surgical ward	41	10.1%
	Recovery	15	3.7%
	Gynecology & obstetric ward	41	10.1%
	Pediatrics ward	32	7.9%
	Operation room	43	10.6%
	Orthopedics	13	3.2%

^*∗*^Outpatient Department.

**Table 2:** Level of compliance with SPs among HCWs working in Gondar University Comprehensive Specialized Hospital, Northwest Ethiopia, 2014 (*n* = 407).

Components of SPs	Level of compliance		
	Always	Sometimes	Seldom
Wash hands before touching a patient	74 (18.2%)	82 (20.1%)	251 (61.7%)
Wash hands before clean or aseptic procedures	161 (39.6%)	144 (35.4%)	102 (25.1%)
Wash hands after body fluid exposure	375 (92.2%)	28 (6.8%)	4 (1%)
Wash hands after touching a patient	113 (27.8%)	124 (30.5%)	170 (41.8%)
Wash hands immediately after removal of gloves	328 (80.6%)	49 (12%)	30 (7.4%)
Wash hands between patient contact	79 (19.4%)	104 (25.6%)	224 (55%)
Wash hands after touching patient surroundings	91 (22.4%)	129 (31.7%)	187 (45.9%)
I provide nursing care considering all patients as potentially infectious	110 (27%)	132 (32.4%)	165 (40.5%)
I protect myself against body fluids of all patients regardless of their diagnosis	132 (32.4%)	151 (37.1%)	122 (30%)
I wear clean gloves whenever there is a possibility of exposure to any body fluids	361 (88.7%)	32 (7.9%)	14 (3.4%)
I change gloves between contacts with different patients	362 (88.9%)	39 (9.5%)	6 (1.5%)
I avoid wearing my gown out of hospital compounds	153 (37.6%)	232 (57%)	22 (5.4%)
I wear a waterproof apron whenever there is a possibility of body fluid splashing in my body	153 (37.6)	211 (51.8%)	43 (10.6%)
I wear eye goggles whenever there is a possibility of body fluid splashing in my face	88 (21.6%)	80 (19.7%)	238 (58.5%)
I sterilize all reusable equipment before being used on another patient	300 (73.7%)	88 (21.6%)	19 (4.7%)
I clean and disinfect equipment and environmental surfaces	156 (38.3%)	148 (36.4%)	103 (25.3%)
I segregate noninfectious wastes in black colour coded dust bin	123 (30.2%)	192 (47.2%)	92 (22.6%)
I segregate infectious medical wastes in yellow coloured coded dust bin	140 (34.4%)	177 (43.5%)	90 (22.1%)
I never bend needles with my hands	311 (76.4%)^*∗*^	68 (16.7%)	28 (6.9%)
I avoid removing used needles from disposable syringes	221 (54.3%)	132 (32.4%)	54 (13.3%)
I place used sharps in puncture-resistant container at point of use	355 (87.2%)	41 (10%)	11 (2.8%)
I never recap needles	239 (58.7%)^*∗*^	100 (24.6%)	68 (16.7%)

^*∗*^Always means never.

**Table 3:** Proportion of HCWs compliance with SPs by profession in Gondar University Comprehensive Specialized Hospital, Northwest Ethiopia, 2014.

Profession	Compliance with SPs			
	Noncompliant		Compliant	
	Frequency	%	Frequency	%
Intern doctors	70	17.2	10	2.5
General practitioners	38	9.3	10	2.5
Anesthetists	13	3.2	1	0.2
Nurses	148	36.4	14	3.4
Midwifes	17	4.2	5	1.2
Laboratory technologists	60	14.7	5	1.2
Radiologists	4	1.0	1	0.2
Physiotherapists	8	2.0	3	0.8
Total	358	88	49	12

**Table 4:** Bivariate and multivariate analysis of factors associated with compliance with SPs among HCWs working in Gondar University Comprehensive Specialized Hospital, Northwest Ethiopia, 2014.

Variable	Compliance with SPs		Crude odds ratio with 95% CI	Adjusted odds ratio with 95% CI
	Noncompliant (*n* = 358)	Compliant (*n* = 49)		
*Sociodemographic and individual factors *				
Sex				
Female	118	26	2.29 (1.26–4.20)	**2.18 **(**1.12–4.23**)
Male	240	23	1	1
Knowledge on SPs				
Unsatisfactory	144	13	1	—
Satisfactory	214	26	1.86 (0.96–3.64)	
Perception of infection risk				
Lower	224	12	1	1
Higher	134	37	5.15 (2.59–10.23)	**3.46 (1.67–7.18)**
*Institutional factors *				
Availability of PPE				
Not readily available	323	37	1	—
Readily available	35	12	2.99 (1.43–6.27)	
Accessibility of PPE				
Not readily accessible	312	28	1	1
Readily accessible	46	21	5.09 (2.67–9.69)	**2.87 (1.41–5.86)**
Management support for safety				
Less frequent	295	24	1	1
More frequent	63	25	4.88 (2.62–9.09)	**2.23 (1.11–4.53)**
Feedback for safety practice				
Less frequent	316	31	1	—
More frequent	42	18	4.37 (2.23–8.49)	
Work place safety climate				
Unsatisfactory	327	36	1	—
Satisfactory	31	13	3.81 (1.83–7.93)	
Training				
No	163	7	1	**1**
Yes	195	42	5.02 (2.19–11.47)	**2.90 (1.20–7.02)**

PPE = personal protective equipment, CI = confidence interval, and SPs = standard precautions.

Hosmer and Lemeshow Test: Chi-square = 3.54, df = 7, and *p* value = 0.83.
